# Effects of cognitive remediation on cognitive dysfunction in partially or fully remitted patients with bipolar disorder: study protocol for a randomized controlled trial

**DOI:** 10.1186/1745-6215-14-378

**Published:** 2013-11-10

**Authors:** Kirsa M Demant, Glennie Marie Almer, Maj Vinberg, Lars Vedel Kessing, Kamilla W Miskowiak

**Affiliations:** 1 Copenhagen Affective Disorder Clinic, Psychiatric Centre Copenhagen, Copenhagen University Hospital, Rigshospitalet, Copenhagen, Denmark

**Keywords:** Affective disorders, Bipolar disorder, Cognitive difficulties, Cognitive dysfunction, Cognitive function, Cognitive remediation, Cognitive training, Computer-assisted cognitive remediation, Occupational function, Psychosocial function

## Abstract

**Background:**

A large proportion of patients with bipolar disorder experience persistent cognitive dysfunction, such as memory, attention and planning difficulties, even during periods of full remission. The aim of this trial is to investigate whether cognitive remediation, a new psychological treatment, improves cognitive function and, in turn, psychosocial function in patients with bipolar disorder in partial or full remission.

**Methods/Design:**

The trial has an evaluator-blind, randomized, between-groups design. Forty patients with bipolar disorder in full or partial remission, aged 18 to 50 years, who report moderate to severe cognitive difficulties, are recruited. Patients are randomized to receive weekly group-based cognitive remediation treatment over 12 weeks in addition to standard treatment or standard treatment alone. Both groups undergo neurocognitive testing and functional magnetic resonance imaging (fMRI) at baseline, post-treatment (week 12) and follow-up (week 26). The primary outcome is improved verbal memory, as measured with the Rey Auditory Verbal Learning Test (RAVLT) from baseline to post-treatment. With inclusion of 40 patients we obtain 86% power to detect a clinically relevant difference in verbal memory between groups. Secondary outcomes are improved attention, executive function and psychosocial function, as measured with the Rapid Visual Information Processing test, the Trail Making Test part B and the Functional Assessment Short Test (FAST), respectively. Tertiary outcomes are improved scores for additional neuropsychological tests of memory, attention, executive function and facial expression recognition, as well as in questionnaires measuring subjective cognitive difficulties, stress, coping strategies, personality traits, depressive symptoms and quality of life.

**Discussion:**

This is the first randomized controlled trial to evaluate the effects of cognitive remediation on cognitive function in patients with bipolar disorder who experience persistent cognitive difficulties despite being in full or partial remission.

**Trial registration:**

ClinicalTrials.gov NCT01457235.

## Background

Worldwide, bipolar disorder (BD) is among the ten leading causes of reduced functional ability [1]. Despite medical treatment with mood stabilizers, newer antipsychotics and antidepressants, a large proportion of patients with BD experience persistent and debilitating cognitive dysfunction [2-4]. In particular, trait-related deficits have been shown in verbal memory, sustained attention and executive function [5,6], as well as in social cognition [7,8]. These cognitive deficits may not simply be side effects of the medical treatment but may also represent a core feature of the psychopathology [9,10]. It has been estimated that 30 to 60% of bipolar patients have trait-related cognitive deficits and that these reduce their occupational and social functioning [11-13] and quality of life [14]. There is an urgent clinical need for new treatment strategies that target cognitive enhancement in BD.

Cognitive remediation (CR) is a new psychological treatment, which aims to improve cognitive function, compensation and coping skills and, consequently, psychosocial function. It has been studied extensively in patients with schizophrenia: a recent meta-analysis of 40 studies demonstrated that CR leads to lasting improvement of cognitive and social function levels in this patient group (effect size = 0.45; 95% confidence interval = 0.31 to 0.59) [15]. Another meta-analysis of 16 studies of CR in mixed groups of patients with affective disorders, affective psychosis, schizophrenia or schizoaffective disorders suggests that patients with BD can achieve beneficial effects of CR that are equivalent to those observed in patients with schizophrenia (effect size = 0.32; 95% confidence interval = 0.20 to 0.43) [16]. Four of these studies included patients with affective disorders; of these, one study in unipolar disorder showed that memory improvement following CR was associated with increased occupational function [17]. Similarly, improved psychosocial and occupational functioning was found in a small, uncontrolled trial of 18 patients with BD [18] and in a small, controlled trial with a mixed sample of patients with unipolar depression and BD [19] with large effect size; (partial *η*^2^ = 0.45). Nevertheless, the studies included in the meta-analysis provide only preliminary evidence.

Recently, a multicentre randomized controlled trial (RCT) investigated the effects of functional remediation versus either psychoeducation or standard care on psychosocial function in euthymic patients with BD [20]. Although patients in the functional remediation group showed functional improvement compared with those in standard care (with small effect size; *d*′ = 0.3), this was not significantly different from the improvement observed in the psychoeducation group. In addition, assessment of cognitive function revealed no beneficial effects of functional remediation over psychoeducation or standard care [20].

The aim of this trial is, therefore, to investigate whether CR has beneficial effects on cognitive and psychosocial function in patients with fully or partially remitted BD who experience persistent cognitive difficulties. The rationale for inclusion of this remitted patient group is that their trait-related cognitive deficits are not successfully targeted by current treatments, as opposed to the state-related deficits in acute affective episodes, which may in some cases resolve fully with affective symptom reduction [2-4]. Beneficial effects on trait-related cognitive deficits would hence point to future implementation of CR for remitted patients with BD, in order to facilitate these patients’ cognitive abilities and capacity to cope with education, work and everyday functioning.

### Hypotheses

The hypotheses of the present study are that CR, in comparison with standard treatment, will:

1. Enhance verbal memory (primary outcome), as measured with the Rey Auditory Verbal Learning Test (RAVLT) from baseline to week 12.

2. Improve sustained attention, executive function and psychosocial function (secondary outcomes), as measured with the Rapid Visual Information Processing, Part B of the Trail Making Test and Functional Assessment Short Test (FAST).

3. Improve additional measures of attention, executive function and facial expression recognition, as measured with a comprehensive neurocognitive test battery; and improve self-reported cognitive and psychosocial function, as measured with questionnaires.

#### *Biomarkers of treatment effects*

In addition, we wish to investigate the following biomarkers of potential beneficial effects of CR on the outcome parameters: plasma levels of brain derived neurotrophic factor (BDNF), stress response, as reflected by morning awakening cortisol levels, and the neuroanatomical underpinnings of memory, attention, executive function and emotional processing. We hypothesize that potential beneficial effects of CR are associated with an increase in levels of plasma BDNF, a reduction in levels of morning awakening cortisol, or changes in the neuroanatomical underpinnings of memory, attention, executive function and emotional processing (see details under section 'Brain biomarkers of potential treatment effects’), or a combination of these.

## Methods/Design

### Study design

Patients will be randomized (1:1) to receive either CR in a group setting or standard treatment in an evaluator-blind between-groups design.

### Participants and screening

Patients will mainly be recruited through the Copenhagen Affective Disorder Clinic, Psychiatric Centre Copenhagen, Copenhagen University Hospital, Rigshospitalet, Copenhagen, Denmark. The centre receives patients with complex affective disorders from the whole capital region. Patients will receive written information about the project and will be given the opportunity to ask questions before deciding whether to participate. Further information is available during the entire course of investigation from any of the researchers involved in the project. Patients will be screened with a medical check and a psychiatric interview using Schedules for Clinical Assessment in Neuropsychiatry [21] to confirm diagnosis, and the 17-item Hamilton Depression Rating Scale, (HDRS-17) [22] and the Young Mania Rating Scale (YMRS) [23] to determine the severity of depressive and manic symptoms.

We will recruit a minimum of 44 patients with the assumption of a 10% dropout rate, leaving us with 40 completers. We include patients with BD (as defined by ICD-10 diagnostic criteria), aged 18 to 50 years, who are in full or partial remission (defined as HDRS-17 and YMRS scores of ≤14) and have subjective complaints of cognitive difficulties of moderate to severe degree (score of ≥4) in at least two of seven cognitive domains on the Massachusetts General Hospital Cognitive and Physical Functioning Questionnaire [24]. Exclusion criteria are: current ECT-treatment, current substance or alcohol abuse, schizophrenia, schizoaffective disorder or significant suicide risk. Patients are permitted to take antidepressants, lithium, antipsychotic medication and benzodiazepines corresponding to <22.5 mg Oxazepam daily.

### Interventions

#### *Cognitive remediation treatment*

Patients randomized to the CR group will receive CR in addition to standard treatment. A group of clinical psychologists at the Copenhagen Affective Disorder Clinic manualized the CR treatment. The CR programme is based on a treatment programme for patients with schizophrenia [25], experience with cognitive rehabilitation in brain damage and thorough clinical and research-based knowledge of cognitive difficulties in BD. Before study initiation, we conducted a small pilot study, including six patients between September and December 2009, with the purpose of testing and revising the treatment manual and session structure and to define treatment length. The CR was then designed to consist of 12 weekly group sessions of two hours each, followed by a booster session four weeks after treatment completion. Groups include between five and eight patients and are run by two group therapists; a specialist in psychiatry with more than 20 years of clinical expertise with bipolar patients and a clinical psychologist trained in neuropsychology.

The programme has three main components: (1) psychoeducation and awareness of cognitive dysfunction in BD, including an emphasis on discovering each patient’s individual profile of cognitive dysfunction; (2) training of compensatory and adaptive strategies in relation to cognitive dysfunction; and (3) computer-assisted cognitive training using RehaCom [26]. The computerized exercises chosen for our intervention target attention and concentration, memory and learning, and executive function. Most exercises are highly ecologically valid as they focus on everyday life situations, thereby optimizing the translation from computer training to real-life cognitive demands, for example, planning activities, shopping for groceries, counting, memorizing short articles or memorizing people’s faces and their names, job titles and phone numbers. The level of difficulty is automatically customized to each patient and increases as the patients’ performance improves. Furthermore, employing computer exercises as part of the treatment provides easily accessible homework and enables an individually tailored training programme to be created despite the group-based intervention.

A number of general principles for the CR sessions were established and are followed by the group therapists to facilitate learning: therapists will give brief lectures on a given topic without the use of PowerPoint slides, to minimize the strain on the patients’ divided attention. To account for the patients’ limited attention spans, the therapists are allowed to talk for a maximum of 5 to 10 minutes without patient involvement, after which patients are required to engage actively in the session. Each session consists of 120 minutes including a 15 minutes break. Homework, which practises attention, memory and executive function, consists of computer training, mindfulness exercises and reading a textbook about cognitive dysfunction in BD. Occasionally, the use of new compensatory and adaptive strategies will be included as part of the homework between sessions. Homework assignments will be discussed in each session along with planning of how the patient can most easily succeed in fulfilling the assignments at home. This includes planning the time of day and for how long each patient will read and complete the assigned computer tasks. For the homework reading tasks, each section of the book is assigned as homework twice, in order to facilitate the application of adaptive and compensatory strategies as well as to enhance the possibility of learning and remembering the contents.

The 12 sessions are divided into four main topics. The first two sessions consist of an introduction to cognitive function and dysfunction and to the principles of CR. In sessions three to five, attention and concentration are the main focus. An emphasis is placed on the importance of attention as a foundation for other areas of cognitive function, such as memory. The main elements in these sessions are psychoeducation on the variation in attentional function in BD, basic neuropsychological knowledge concerning various aspects of attentional function, computer training, and compensatory and adaptive strategies. Examples of such strategies for enhancing attentional function are reading with and without background music in order to discover what works best, allowing frequent breaks during attention straining tasks, such as setting an alarm when reading, and limiting oneself to carry out one task at a time rather than attempt to multitask a number of activities or initiate new activities before other activities are completed. Finally, therapists train patients in mindfulness meditation as a means to enhance attentional capacity [27], and in subsequent sessions, a brief mindfulness meditation will be carried out at the beginning of each session.

Sessions six to eight address memory and learning. During these sessions, various exercises on remembering visual and auditory verbal input are performed. These exercises include a reading task where patients are handed a brief newspaper article on a relevant topic ten minutes before the 15 minutes break during a session. The timing is chosen to ensure a small time gap between encoding and recall of the content of the article. Patients are asked to read the article with the intention of giving a brief resume in front of the rest of the group after the break. Before commencing the task, we refresh memory techniques, such as reading aloud, taking brief notes and using visual imagery in order to remember key points. Another exercise is a conversational task, also carried out just before the 15 minutes break during sessions. In this exercise, patients work in pairs; one of them pretending to be a close friend or relative and the other asking questions to get to know this person. Again, following the 15 minutes break they are asked to tell the rest of the group about the person they had 'interviewed’. The idea of pretending to be another person is to ensure that the listening patient does not have prior knowledge of the conversation partner.

Brief talks are given on various aspects of memory, such as long-term versus short-term memory and working memory, and verbal versus visuospatial memory. An introduction to establishing structure in everyday life is followed by homework on this topic. Homework includes establishing a routine of using only one calendar or diary, whether it is an electronic version or a traditional paper version, rather than several, which may cause confusion. Furthermore, patients are instructed in efficient use of a diary or calendar by including all appointments plus time for rest and physical exercise, grocery shopping, and so on. Possible cognitive side effects of medication and substance abuse are discussed.

Sessions 9 to 12 are dedicated to executive function in everyday life. Thorough discussions of where executive dysfunction arises provide a foundation for individualized homework assignments in order to improve these areas of functioning. In the sessions, we carry out troubleshooting as a group-based exercise. One patient mentions a problem, such as being incapable of cooking a meal or always being late for appointment and feeling stressed as a result. With thorough guidance from the group therapists, we break down the problem into as many subcomponents as possible and try to conjure up new and creative solutions to try out as homework in between sessions. The homework assignments are followed up in great detail. Also, at this point in the treatment programme, reading of fiction as cognitive training of attention and verbal memory is introduced. Each patient is encouraged to choose a book as a homework assignment and a detailed plan for the reading process is established. An emphasis is placed on making the treatment programme as individualized as possible.

#### *Standard treatment group*

Patients in the control group receive standard treatment consisting of the standard out-patient mental health services routines in the capital region of Denmark either at the Copenhagen Affective Disorder Clinic, community psychiatric centres or private specialists in psychiatry. Standard treatment involves psychopharmacological mood stabilization and relapse prevention and, in our clinic and most community psychiatric centres, psychoeducation. In contrast to the intervention group, standard treatment does not involve any specific cognitive training.

### Randomization

Pharma Consulting Group [28] performed the randomization of the patients with stratification for age (<35 years) and years of education (<15 years).

### Blinding

Study personnel involved in the evaluation of outcomes are blinded to treatment allocation. Blinding is maintained throughout the study, data management, outcome assessment and data analysis.

### Outcome assessments

The main study outcomes (cognitive and psychosocial function) and biomarkers of potential treatment effects (plasma BDNF, cortisol levels and neuroanatomical underpinnings) will be measured at baseline, after completion of treatment (week 12) and at follow-up (week 26). We also rate affective symptoms at these three time points and additionally at weeks 6 and 20 to uncover potential mood swings in the periods between outcome assessments.

#### *Neurocognitive function*

Patients undergo neurocognitive testing with a comprehensive test battery including the following RAVLT subtests: total recall across the five learning trials (I to V), RAVLT recall following interference (trial VI), recall following a 30 minute delay and recognition from baseline to week 12 [29,30]; Trail Making Test parts A and B [31]; Repeatable Battery of the Assessment of Neuropsychological Status digit span and coding [32]; WAIS-III Letter-Number Sequencing [33]; Verbal and Semantic Fluency Tests [34]; Danish Adult Reading Test [35]; the computerized Facial Expression Recognition Task test from the Oxford Emotional Test Battery (P1Vital; Oxford); and the following computerized tests from Cambridge Cognition (CANTAB): Simple Reaction Time; Rapid Visual Information Processing; Delayed Match-to-Sample; and Spatial Working Memory.

#### *Psychosocial function*

Psychosocial function will be measured with FAST [36], which is specifically developed and validated for BD.

#### *Questionnaires*

Self-reported cognitive difficulties, stress, coping strategies, personality traits, depressive symptoms and quality of life are measured using the following questionnaires: Massachusetts General Hospital Cognitive and Physical Functioning Questionnaire; State-Trait Anxiety Inventory [37]; Eysenck Personality Questionnaire [38]; Coping Inventory for Stressful Situations [39]; Language and Words; Cognitive Failures Questionnaire [40]; World Health Organization Quality of life BREF [41]; Cohen’s Perceived Stress Scale [42]; Positive and Negative Affect Scales [43]; European Quality of Life-5 Dimensions [44]; Beck Depression Inventory [45] and Work and Social Adjustment Scale [46].

#### *Brain biomarkers of potential treatment effects*

The neuroanatomical underpinnings of potential beneficial cognitive effects of CR are explored with functional and structural magnetic resonance imaging (fMRI and MRI) at baseline and weeks 12 and 26. In particular, we will investigate whether CR modulates neural responses underlying memory, executive function and social cognition with a picture encoding task (revised from Miskowiak *et al*. [47]), an *N*-back (2-back versus 0-back) working memory task, and an emotional face processing task using fearful and happy NimStim faces. We hypothesize that the CR group compared with the standard treatment group will (1) increase memory-related activation in medial temporal regions including the hippocampus and that this will correlated with memory performance; (2) enhance neural response in a fronto-parietal network during the 2-back task, which is associated with improved executive function; and (3) modulate neural responses to emotional faces in a fronto-limbic circuitry, which is accompanied by improved facial expression recognition. In addition, we will explore whether CR induces changes in hippocampus structure, white matter fibre structure and brain connectivity.

#### *Imaging data analysis*

Both fMRI and MRI data are collected with a Siemens Trio scanner operating at 3.0 T at the Danish Research Centre for Magnetic Resonance, Hvidovre Hospital, Denmark. Image analysis is carried out using tools from FMRIB Software Library [48,49]. BET is used for brain extraction of the T1-weighted images and FLIRT is used to perform affine alignment of the diffusion and T1-weighted images. The FIRST tool, which is part of FMRIB’s Software Library version 5.0 [48], is used to segment the hippocampus automatically for analyses of changes in volume and shape. The fMRI data from the memory and emotion processing tasks will preprocessed and analyzed using FMRIB Expert Analysis Tool version 6.0, part of FMRIB Software Library, whilst fMRI data from the *N*-back task will preprocessed and analyzed using Statistical Parametric Mapping software version 8. Region-of-interest analyses of prefrontal, limbic and parietal regions, as well as exploratory whole-brain analyses will be performed. Diffusion images will be analyzed using tools from FMRIB Diffusion Toolkit. Probabilistic modelling of diffusion parameters and tractography are carried out using previously described methods [50].

In particular, we will compare differences in neural activity from baseline to post-treatment between CR and standard treatment groups for patients who have completed both baseline and post-treatment scanning sessions. In addition, cross-sectional post-treatment comparisons between the groups will be made in the event of missing data at baseline. Pearson’s coefficients are calculated to determine the relationship between MRI changes and neuropsychological improvements.

### Sample size and power calculation

Test sample and statistical power is calculated by nQuery Advisor 5.0 software. The primary outcome is a change in verbal memory measured with RAVLT from baseline to week 12 between CR and standard treatment groups. A recent study demonstrated that the average California Verbal Learning Test (a verbal learning test equivalent to the RAVLT) total recall score for patients with remitted BD is 52.0, whilst the score for healthy age-matched control subjects was 60.7 (out of a maximum 75) [51]. Based on this, we would expect a clinically relevant difference in the change between groups to be at least 4 points in RAVLT total recall. Assuming an average RAVLT total recall score of 56 subsequent to CR and 52 (that is, a difference in the change of 4 points) subsequent to standard treatment and a standard deviation of 4 points for both groups, a sample size of *N* = 40 patients (*N* = 20 per group) will achieve a statistical power of 86% to demonstrate a clinically relevant verbal memory improvement with CR versus standard treatment.

### Statistical analyses of primary, secondary and tertiary outcomes

To investigate the effects of CR versus standard treatment from baseline to week 12 (primary outcome assessment time), we will use repeated measures analysis of covariance with adjustment for stratification variables, and with and without adjustment for mood changes from baseline to week 12. To investigate long-term effects of CR versus standard treatment in week 26, we will implement a linear mixed-effects model with random intercept for each participant, structured as a two-level model specifying a correlation of samples within participants, with adjustment for stratification variables, and with and without adjustments for mood changes. Significant interactions will be analyzed further with simple main effect analyses. All statistical analyses of behavioural data, mood ratings and questionnaires will be performed using the Statistical Package for Social Sciences.

### Ethical considerations

The study is approved by The Regional Committee on Biomedical Research Ethics (protocol number H-1-2010-039), The Danish Data Protection Agency (protocol number 2010-41-4710) and ClinicalTrials.gov (identifier NCT01457235).

## Discussion

This trial is the first RCT to investigate the effects of CR on cognitive function in BD. Data from patients with schizophrenia and preliminary results from small exploratory studies in mixed groups of patients with affective disorders suggest beneficial effects [16-19], although a recent study of functional remediation in BD showed no significant effect on cognition [20]. The present trial therefore serves to meet the clinical need for more specific and thorough investigations of the effects of CR on patients’ cognitive function.

### Limitations

CR in a group setting has been investigated in only a few studies [15,20] and it is unclear whether group therapy is less appealing than individual therapy for patients with cognitive difficulties. It is, however, our experience from the pilot study and patient feedback from the present trial that patients benefit from group-based training; it is often easier for patients to acknowledge and accept their own cognitive difficulties and to learn to cope with them when they are aware of other patient’s problems and improvements. Another limitation is that it is unclear how many training sessions are optimal for bipolar patients with cognitive dysfunction. In an uncontrolled study by Deckersbach *et al*. [18], 14 individual sessions of CR were provided and results showed lower residual symptoms and increased occupational and overall psychosocial functioning in patients with BD. Moreover, improvements in executive functioning were associated with improved occupational functioning. Studies of patients with schizophrenia have typically included an average treatment length of 32.2 hours (range = 4 to 130 hours), provided over 16.7 weeks (range = 2 to 104 weeks) with a therapy intensity of 2.2 sessions per week (range = 0.6 to 5 sessions per week) [15].

We chose to provide 12 + 1 sessions based on the experience from our pilot study that this length is sufficient for improvement in most patients. However, if this first exploratory study finds an effect of CR on cognition, future studies are warranted to explore the optimal length of treatment and frequency of CR sessions. Finally, the choice of a good control group is often complicated in psychological intervention studies. We chose to compare CR as an add-on to standard treatment with standard treatment alone. With such a design we cannot be sure that a potential beneficial effect of the active intervention is due specifically to the CR program or to an unspecific effect of solely taking part in group sessions. Colom *et al*. [52] used an elegant, if time- and resource-consuming, design by comparing group psychoeducation with unspecific group intervention in their seminal paper aiming to detect the specific effects of psychoeducation. It can be argued whether the interaction between patients in the unspecific group intervention is comparable to part of the CR treatment. Using our design, the entire combined effect of CR and group sessions, including the interaction with other patients with similar problems in group sessions, is investigated.

### Advantages

RAVLT was chosen as the primary outcome measure for the following reasons: (a) remitted patients with BD show deficits on this test [53,54]; (b) it is a standardized, internationally accepted and valid test of verbal memory [55]; (c) there is a high correlation between verbal memory and psychosocial function [56], which makes it clinically relevant; and (d) other RCTs have found significant effects of CR on verbal memory in patients with schizophrenia [57-59]. However, there is also consistent evidence for trait-related deficits in sustained attention in BD and negative effects of this on psychosocial function [56]. In addition, CR has been shown to improve attention in schizophrenia [57,59] and unipolar disorder [17], and both verbal memory and sustained attention are addressed in our CR programme. Therefore, the priority of verbal memory over sustained attention as the primary outcome may be somewhat arbitrary.

The study has a naturalistic approach with few exclusion criteria. Patients should have BD, should be in complete or partial remission and should self-report cognitive difficulties. To make treatment with CR meaningful, patients with current substance or alcohol abuse or significant suicide risk are excluded but standard psychopharmacological treatment is allowed. Consequently, if CR is found to produce beneficial effects, the intervention can be implemented to treat all bipolar patients in complete or partial remission with subjective complaints of cognitive difficulties. We expect a high patient acceptance and adherence as the CR program is easy to follow and not too time consuming and also because of the supportive positive effects from the group setting in itself. From a feasibility perspective, the intervention is relatively inexpensive as two therapists in our affective disorder clinic deliver the group sessions. Although the CR intervention is standardized according to a manual and the computer training software [26] it is likely that treatment with CR is best offered in specialized affective disorder clinics with expertise within pharmacological and psychological treatment of BD.

### Perspectives

If CR proves effective, it can easily be implemented in future treatment of BD to facilitate patients’ cognitive function and thereby potentially improve their psychosocial function.

## Trial status

Patient enrolment started in September 2011 and is ongoing until a minimum 40 patients have completed assessment at week 12 (primary outcome assessment time).

## Abbreviations

BD: Bipolar disorder; BDNF: Brain derived neurotrophic factor; CR: Cognitive remediation; FAST: Functional Assessment Short Test; fMRI: Functional magnetic resonance imaging; HDRS-17: 17-item Hamilton Depression Rating Scale; MRI: Magnetic resonance imaging; RAVLT: Rey Auditory Verbal Learning Test; RCT: Randomized controlled trial; YMRS: Young Mania Rating Scale.

## Competing interests

LVK has been a consultant for Lundbeck and AstraZenica within the last three years. MV has been a consultant for Eli Lilly, Lundbeck, AstraZeneca and Servier. KWM has been a consultant for Lundbeck. KMD and GMA have no competing interests.

## Authors’ contributions

KWM, MV and LVK conceived the trial. KWM authored the first draft of the trial protocol, which was then revised by LVK and MV. KMD and LVK authored the first version of this article, GMA authored the CR intervention part and MV and KWM revised and optimized the article. All authors read and approved the final manuscript.

## References

[B1] Lopez AD Murray CC The global burden of disease, 1990–2020 Nat Med 1998 4 11 1241 1243 10.1038/3218 9809543

[B2] Chamberlain SR Sahakian BJ Cognition in mania and depression: psychological models and clinical implications Curr Psychiatry Rep 2004 6 6 451 458 10.1007/s11920-004-0010-3 15538994

[B3] Chamberlain SR Sahakian BJ The neuropsychology of mood disorders Curr Psychiatry Rep 2006 8 6 458 463 10.1007/s11920-006-0051-x 17162825

[B4] Martinez-Aran A Vieta E Colom F Reinares M Benabarre A Gasto C Salamero M Cognitive dysfunctions in bipolar disorder: evidence of neuropsychological disturbances Psychother Psychosom 2000 69 1 2 18 10.1159/000012361 10601830

[B5] Goldberg JF Chengappa KN Identifying and treating cognitive impairment in bipolar disorder Bipolar Disord 2009 11 Suppl 2 123 137 19538691 10.1111/j.1399-5618.2009.00716.x

[B6] Burdick KE Goldberg JF Harrow M Faull RN Malhotra AK Neurocognition as a stable endophenotype in bipolar disorder and schizophrenia J Nerv Ment Dis 2006 194 4 255 260 10.1097/01.nmd.0000207360.70337.7e 16614546

[B7] Bozikas VP Tonia T Fokas K Karavatos A Kosmidis MH Impaired emotion processing in remitted patients with bipolar disorder J Affect Disord 2006 91 1 53 56 10.1016/j.jad.2005.11.013 16413611

[B8] Getz GE Shear PK Strakowski SM Facial affect recognition deficits in bipolar disorder J Int Neuropsychol Soc 2003 9 4 623 632 12755174 10.1017/S1355617703940021

[B9] Arts B Jabben N Krabbendam L Van OJ A 2-year naturalistic study on cognitive functioning in bipolar disorder Acta Psychiatr Scand 2011 123 3 190 205 10.1111/j.1600-0447.2010.01601.x 20846251

[B10] Lopez-Jaramillo C Lopera-Vasquez J Ospina-Duque J Garcia J Gallo A Cortez V Palacio C Torrent C Martinez-Aran A Vieta E Lithium treatment effects on the neuropsychological functioning of patients with bipolar I disorder J Clin Psychiatry 2010 71 8 1055 1060 10.4088/JCP.08m04673yel 20361895

[B11] Torrent C Martinez-Aran A Del Mar BC Reinares M Daban C Sole B Rosa AR Tabares-Seisdedos R Popovic D Salamero M Vieta E Long-term outcome of cognitive impairment in bipolar disorder J Clin Psychiatry 2012 73 7 e899 e905 10.4088/JCP.11m07471 22901360

[B12] Martinez-Aran A Vieta E Reinares M Colom F Torrent C Sanchez-Moreno J Benabarre A Goikolea JM Comes M Salamero M Cognitive function across manic or hypomanic, depressed, and euthymic states in bipolar disorder Am J Psychiatry 2004 161 2 262 270 10.1176/appi.ajp.161.2.262 14754775

[B13] Martino DJ Strejilevich SA Scapola M Igoa A Marengo E Ais ED Perinot L Heterogeneity in cognitive functioning among patients with bipolar disorder J Affect Disord 2008 109 1–2 149 156 18234352 10.1016/j.jad.2007.12.232

[B14] Brissos S Dias VV Carita AI Martinez-Aran A Quality of life in bipolar type I disorder and schizophrenia in remission: clinical and neurocognitive correlates Psychiatry Res 2008 160 1 55 62 10.1016/j.psychres.2007.04.010 18485488

[B15] Wykes T Huddy V Cellard C McGurk SR Czobor P A meta-analysis of cognitive remediation for schizophrenia: methodology and effect sizes Am J Psychiatry 2011 168 5 472 485 10.1176/appi.ajp.2010.10060855 21406461

[B16] Anaya C Martinez AA Ayuso-Mateos JL Wykes T Vieta E Scott J A systematic review of cognitive remediation for schizo-affective and affective disorders J Affect Disord 2012 142 1–3 13 21 22840620 10.1016/j.jad.2012.04.020

[B17] Elgamal S McKinnon MC Ramakrishnan K Joffe RT MacQueen G Successful computer-assisted cognitive remediation therapy in patients with unipolar depression: a proof of principle study Psychol Med 2007 37 9 1229 1238 10.1017/S0033291707001110 17610766

[B18] Deckersbach T Nierenberg AA Kessler R Lund HG Ametrano RM Sachs G Rauch SL Dougherty D RESEARCH: cognitive rehabilitation for bipolar disorder: an open trial for employed patients with residual depressive symptoms CNS Neurosci Ther 2010 16 5 298 307 10.1111/j.1755-5949.2009.00110.x 19895584 PMC2888654

[B19] Naismith SL Redoblado-Hodge MA Lewis SJ Scott EM Hickie IB Cognitive training in affective disorders improves memory: a preliminary study using the NEAR approach J Affect Disord 2010 121 3 258 262 10.1016/j.jad.2009.06.028 19616856

[B20] Torrent C Bonnin C del M Martínez-Arán A Valle J Amann BL González-Pinto A Crespo JM Ibáñez Á Garcia-Portilla MP Tabarés-Seisdedos R Arango C Colom F Solé B Pacchiarotti I Rosa AR Ayuso-Mateos JL Anaya C Fernández P Landín-Romero R Alonso-Lana S Ortiz-Gil J Segura B Barbeito S Vega P Fernández M Ugarte A Subirà M Cerrillo E Custal N Menchón JM Efficacy of functional remediation in bipolar disorder: a multicenter randomized controlled study Am J Psychiatry 2013 170 852 859 10.1176/appi.ajp.2012.12070971 23511717

[B21] Wing JK Babor T Brugha T Burke J Cooper JE Giel R Jablenski A Regier D Sartorius N SCAN: schedules for clinical assessment in neuropsychiatry Arch Gen Psychiatry 1990 47 6 589 593 10.1001/archpsyc.1990.01810180089012 2190539

[B22] Hamilton M A rating scale for depression J Neurol Neurosurg Psychiatry 1960 23 56 62 10.1136/jnnp.23.1.56 14399272 PMC495331

[B23] Young RC Biggs JT Ziegler VE Meyer DA A rating scale for mania: reliability, validity and sensitivity Br J Psychiatry 1978 133 429 435 10.1192/bjp.133.5.429 728692

[B24] Fava M Graves LM Benazzi F Scalia MJ Iosifescu DV Alpert JE Papakostas GI A cross-sectional study of the prevalence of cognitive and physical symptoms during long-term antidepressant treatment J Clin Psychiatry 2006 67 11 1754 1759 10.4088/JCP.v67n1113 17196056

[B25] Wykes T Reeder C Landau S Everitt B Knapp M Patel A Romeo R Cognitive remediation therapy in schizophrenia: randomised controlled trial Br J Psychiatry 2007 190 421 427 10.1192/bjp.bp.106.026575 17470957

[B26] Hasomed DE RehaCom [http://www.hasomed.de/en/products/rehacom-cognitive-therapy.html]

[B27] Chiesa A Calati R Serretti A Does mindfulness training improve cognitive abilities? A systematic review of neuropsychological findings Clin Psychol Rev 2011 31 3 449 464 10.1016/j.cpr.2010.11.003 21183265

[B28] Pharma Consulting Group [http://pharmaconsultinggroup.com/]

[B29] Rey A Psychological examination of traumatic encephalopathy Archives de Psychologie 1941 28 286 340

[B30] Rey A L’examen clinique en psychologie [Clinical tests in psychology] 1964 Paris: Presses Universitaires de France

[B31] Army Individual Test Battery Manual of Directions and Scoring 1944 Washington, DC: War Department, Adjutant General’s Office

[B32] Randolph C RBANS Manual: Repeatable Battery for the Assessment of Neuropsychological Status 1998 San Antonio, TX: The Psychological Corporation

[B33] Wechsler D Wechsler Adult Intelligence Scale-III 1997 San Antonio: The Psychological Corporation

[B34] Borkowski JG Benton AL Spreen O Word fluency and brain damage Neuropsychologia 1967 5 135 140 10.1016/0028-3932(67)90015-2

[B35] Nelson HE O’Connell A Dementia: the estimation of premorbid intelligence levels using the new adult reading test Cortex 1978 14 2 234 244 10.1016/S0010-9452(78)80049-5 679704

[B36] Rosa AR Sanchez-Moreno J Martinez-Aran A Salamero M Torrent C Reinares M Comes M Colom F Van RW Ayuso-Mateos JL Kapczinski F Vieta E Validity and reliability of the functioning assessment short test (FAST) in bipolar disorder Clin Pract Epidemiol Ment Health 2007 3 5 10.1186/1745-0179-3-5 17555558 PMC1904447

[B37] Spielberger CD State-Trait Anxiety Inventory: Bibliography 1989 2 Palo Alto, CA: Consulting Psychologists Press

[B38] Eysenck HJ Eysenck SBG Manual of the Eysenck Personality Questionnaire 1975 London: Hodder and Stoughton

[B39] Endler NS Parker JDA Coping Inventory for Stressful Situations (CISS): Manual 1990 Toronto: Multi-Health Systems

[B40] Broadbent DE Cooper PF FitzGerald P Parkes KR The cognitive failures questionnaire (CFQ) and its correlates Br J Clin Psychol 1982 21 Pt 1 1 16 7126941 10.1111/j.2044-8260.1982.tb01421.x

[B41] Group WHOQOL The Development of the World Health Organization Quality of Life Assessment Instrument (the WHOQOL) 1994 Berlin: Springer

[B42] Cohen S Kamarck T Mermelstein R A global measure of perceived stress J Health Soc Behav 1983 24 4 385 396 10.2307/2136404 6668417

[B43] Watson D Clark LA Tellegen A Development and validation of brief measures of positive and negative affect: the PANAS scales J Pers Soc Psychol 1988 54 6 1063 1070 3397865 10.1037//0022-3514.54.6.1063

[B44] The EuroQol Group EuroQol – a new facility for the measurement of health-related quality of life Health Policy 1990 16 3 199 208 10109801 10.1016/0168-8510(90)90421-9

[B45] Beck AT Ward CH Mendelson M Mock J Erbaugh J An inventory for measuring depression Arch Gen Psychiatry 1961 4 561 571 10.1001/archpsyc.1961.01710120031004 13688369

[B46] Mundt JC Marks IM Shear MK Greist JH The work and social adjustment scale: a simple measure of impairment in functioning Br J Psychiatry 2002 180 461 464 10.1192/bjp.180.5.461 11983645

[B47] Miskowiak K O’Sullivan U Harmer CJ Erythropoietin enhances hippocampal response during memory retrieval in humans J Neurosci 2007 27 11 2788 2792 10.1523/JNEUROSCI.5013-06.2007 17360900 PMC6672568

[B48] FMRIB Software Library [http://fsl.fmrib.ox.ac.uk/fsl/fslwiki/]

[B49] Smith S Jenkinson M Woolrich M Beckmann C Behrens T Johansen-Berg H Bannister P De Luca M Drobnjak I Flitney D Niazy R Saunders J Vickers J Zhang Y De Stefano N Brady J Matthews P Advances in functional and structural MR image analysis and implementation as FSL Neuroimage 2004 23 S1 208 219 10.1016/j.neuroimage.2004.07.051 15501092

[B50] Behrens TE Berg HJ Jbabdi S Rushworth MF Woolrich MW Probabilistic diffusion tractography with multiple fibre orientations: what can we gain? Neuroimage 2007 1;34 1 144 155 10.1016/j.neuroimage.2006.09.018 PMC7116582 17070705

[B51] Smith DJ Muir WJ Blackwood DH Neurocognitive impairment in euthymic young adults with bipolar spectrum disorder and recurrent major depressive disorder Bipolar Disord 2006 8 1 40 46 10.1111/j.1399-5618.2006.00275.x 16411979

[B52] Colom F Vieta E Martinez-Aran A Reinares M Goikolea JM Benabarre A Torrent C Comes M Corbella B Parramon G Corominas J A randomized trial on the efficacy of group psychoeducation in the prophylaxis of recurrences in bipolar patients whose disease is in remission Arch Gen Psychiatry 2003 60 4 402 407 10.1001/archpsyc.60.4.402 12695318

[B53] Robinson LJ Thompson JM Gallagher P Goswami U Young AH Ferrier IN Moore PB A meta-analysis of cognitive deficits in euthymic patients with bipolar disorder J Affect Disord 2006 93 1–3 105 115 16677713 10.1016/j.jad.2006.02.016

[B54] Robinson LJ Ferrier IN Evolution of cognitive impairment in bipolar disorder: a systematic review of cross-sectional evidence Bipolar Disord 2006 8 2 103 116 10.1111/j.1399-5618.2006.00277.x 16542180

[B55] Macartney-Filgate MS Vriezen ER Intercorrelation of clinical tests of verbal memory Arch Clin Neuropsychol 1988 3 2 121 126 14591264

[B56] Depp CA Mausbach BT Harmell AL Savla GN Bowie CR Harvey PD Patterson TL Meta-analysis of the association between cognitive abilities and everyday functioning in bipolar disorder Bipolar Disord 2012 14 3 217 226 10.1111/j.1399-5618.2012.01011.x 22548895 PMC3396289

[B57] D’Amato T Bation R Cochet A Jalenques I Galland F Giraud-Baro E Pacaud-Troncin M Augier-Astolfi F Llorca PM Saoud M Brunelin J A randomized, controlled trial of computer-assisted cognitive remediation for schizophrenia Schizophr Res 2011 125 2–3 284 290 21094025 10.1016/j.schres.2010.10.023

[B58] McGurk SR Mueser KT DeRosa TJ Wolfe R Work, recovery, and comorbidity in schizophrenia: a randomized controlled trial of cognitive remediation Schizophr Bull 2009 35 2 319 335 10.1093/schbul/sbn182 19269925 PMC2659315

[B59] Grynszpan O Perbal S Pelissolo A Fossati P Jouvent R Dubal S Perez-Diaz F Efficacy and specificity of computer-assisted cognitive remediation in schizophrenia: a meta-analytical study Psychol Med 2011 41 1 163 173 10.1017/S0033291710000607 20380784

